# Expression of Sucrose Transporters from *Vitis vinifera* Confer High Yield and Enhances Drought Resistance in Arabidopsis

**DOI:** 10.3390/ijms21072624

**Published:** 2020-04-09

**Authors:** Yumeng Cai, Jing Yan, Wenrui Tu, Zhefang Deng, Wenjie Dong, Han Gao, Jinxu Xu, Nan Zhang, Ling Yin, Qingyong Meng, Yali Zhang

**Affiliations:** 1 Beijing Advanced Innovation Center for Food Nutrition and Human Health, College of Food Science and Nutritional Engineering, China Agricultural University, Beijing 100083, China; caiyumeng917@163.com (Y.C.); yanjingjing1618@163.com (J.Y.); twr6637@163.com (W.T.); 531128763@163.com (Z.D.); dongwenjie1134@163.com (W.D.); fadaiclub@126.com (H.G.); xujinxujason@gmail.com (J.X.); zhangnancau@163.com (N.Z.); 2 Tianjin Key Laboratory of Crop Genetics and Breeding, Crops Research Institute, Tianjin Academy of Agricultural Sciences, Tianjin 300384, China; 3 Guangxi Crop Genetic Improvement and Biotechnology Key Lab, Guangxi Academy of Agricultural Sciences, Nanning 530007, China; yinling1985@gmail.com; 4 Beijing Advanced Innovation Center for Food Nutrition and Human Health, College of Biological Science, China Agricultural University, Beijing 100193, China; qymeng@cau.edu.cn; 5 The State Key Laboratory for Agrobiotechnology, College of Biological Sciences, China Agricultural University, Beijing 100193, China

**Keywords:** *Vitis vinifera*, VvSUC, overexpression, Arabidopsis, srong phenotype, high production, drought resistance

## Abstract

Sucrose is the predominant form of sugar transported from photosynthetic (source) to non-photosynthetic (sink) organs in higher plants relying on the transporting function of sucrose transporters (SUTs or SUCs). Many SUTs have been identified and characterized in both monocots and dicots. However, the function of sucrose transporters (SUTs or SUCs) from *Vitis* is not clear. As the world’s most planted grape species, *Vitis vinifera* owns three sucrose transport activity verified SUTs. In this study, we constructed three kinds of *VvSUC (Vitis vinifera SUC)*-overexpressing transgenic Arabidopsis. *VvSUC*-overexpressing transgenic Arabidopsis was cultured on sucrose-supplemented medium. *VvSUC11*- and *VvSUC12*-overexpressing lines had similar thrived growth phenotypes, whereas the size and number of leaves and roots from *VvSUC27*-overexpressing lines were reduced compared with that of WT. When plants were cultured in soil, all SUT transgenic seedlings produced more number of leaves and siliques, resulting in higher yield (38.6% for *VvSUC12*-transformants) than that of WT. Besides, *VvSUC27*-transformants and *VvSUC11*-transformants enhanced drought resistance in Arabidopsis, providing a promising target for crop improvement

## 1. Introduction

Carbon fixation occurs in photosynthetic (source) organs, such as mature leaves. Carbon fixed in excess during the day can be exported as sucrose to the non-photosynthetically heterotrophic (sink) organs, such as roots, developing leaves, reproductive organs, seeds, the shoot apex, tubers and meristems [1]. Therefore, the function of sucrose transporters (SUTs or SUCs) in the translocation of sucrose is indispensable for plant development. Since the discovery of spinach SoSUT1 [2], several other SUTs have been identified and characterized in both monocots and dicots [3,4,5,6,7]. However, not all SUT family members are well characterized and our understanding on the function of SUTs in *Vitis* is limited.

Phylogenetic analysis has revealed that there are five independent SUT subfamilies that make up the type SUT1-SUT5 clades [8], and dicotyledonous transporters belong to the SUT1, SUT2/SUC3, and SUT4 subfamilies. Most members of the SUT1 clade are high affinity Suc/proton cotransporters that are mostly located on the plasma membranes of the sieve element (SE) [9,10,11], companion cells (CC) [8], or both cell types [12]. SUT1 is mainly expressed in sucrose-exporting source leaves [11]. Similarly, LeSUT1 is the main phloem loader of sucrose in *Solanaceae* [10].

Most members of the SUT2/SUC3 and SUT4 subfamilies have been reported to be low-affinity sucrose transporters. The SUT2/SUC3 clades are structurally different from other SUT clades as they have extended domains at their N-termini and elongated central cytoplasmic loops [13]. The SUT2/SUC3 transporters are localized to SE plasma membranes [10,11,14,15], except ZmSUT2, which is on the vacuolar membrane. ZmSUT2 can remobilize sucrose from the vacuole, which contributes to maize development and agronomic yield [16]. SUT2/SUC3 is reported to be expressed predominantly in the sink organs, such as sink leaves, stems, and fruits, whereas LeSUT2 and AtSUC3 (AtSUT2) are colocalized in the SEs [11].

The SUT4 subfamilies have been mostly identified in diverse subcellular localizations and with diverse functions; for example, AtSUT4, LeSUT4, and StSUT4 are localized to enucleated SEs in plants [17]. StSUT4 is located on the plasma membrane, mainly in the sink leaves, of young potato plantlets and at the perinuclear ring, which could affect flowering, tuberization, shade-avoidance response, circadian-regulated genes, and ethylene production [7,18]. AtSUT4 has been identified in the vacuole, suggesting that it is involved in the transport and vacuolar storage of sucrose [19].

Multiple SUT isoforms are responsible for loading phloem, transporting and unloading sucrose; however, the SUT clades in vine species have not been well characterized with respect to SUT functional diversity. *Vitis* as a genus has about 60 vining species. *Vitaceae* species are used as medicinal herbs and wine making worldwide [20]. The type and concentration of sugars and acids in grapes contribute to the organoleptic quality of the berries, the flavor and stability of wine [21]. Sucrose is the predominant form of sugar transportation in grapevine; therefore, the function of *Vitis vinifera* SUCs (VvSUCs) are important. Although the three functional SUTs—VvSUC11, VvSUC12, and VvSUC27—have been identified in grape berries [22], differences in the function of these SUTs in *V. vinifera* is not well understood. As a member of the SUT1 subfamily, VvSUC27 has been described as a low-affinity/high-capacity SUT, VvSUC11 has been characterized as a member of the traditional low-affinity SUT4 subfamily, while VvSUC12 belongs to the SUT2/SUC3 subfamily [23,24,25,26]. Previously, we have elucidated that VvSUC27 is located on the plasma membrane and negatively correlated with sugar accumulation, which indicates its important role in plant development [27]. In this study, the functions of SUTs were further systematically evaluated using transgenic *Arabidopsis thaliana* lines.

## 2. Results

### 2.1. Amino Acids Sequences Analysis of VvSUC Proteins

We initially studied the phylogenetic relationships between SUT sequences in *Arabidopsis thaliana*, *Lycopersicon esculentum*, and *Vitis vinifera* “Thompson Seedless” (Figure S1). The SUT sequences in *V. vinifera* can be classified into three broad classes and share 40.95% to 51.61% similarity between each other (Table S1).

### 2.2. SUT Overexpression Alters Morphological Phenotypes and the Germination Rate of Arabidopsis Seeds

**Figure 1:**
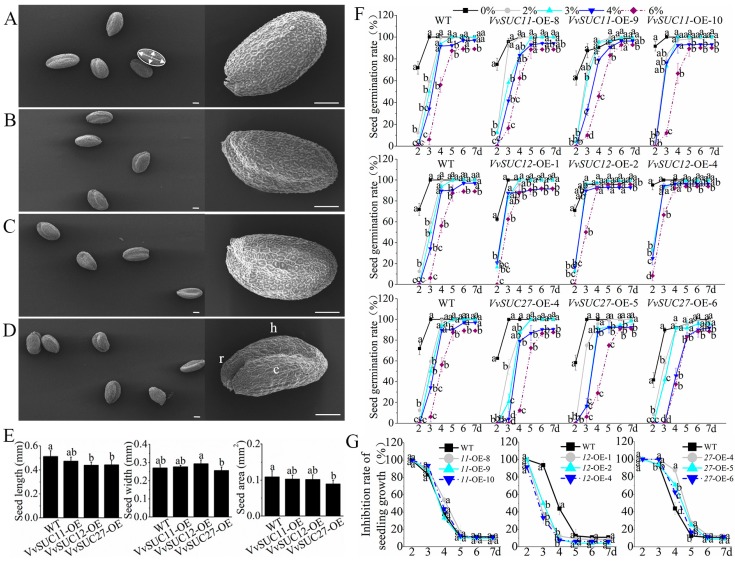
Morphological phenotypes of seeds and the effects of sucrose concentration on the seed germination of *Vitis vinifera* sucrose transporter (*VvSUC*)-overexpressing (OE) Arabidopsis. (**A**) to (**D**) morphological phenotypes were observed by scanning electron microscopy in the (**A**) wild type (WT), (**B**) *VvSUC11*-, (**C**) *VvSUC12*-, and (**D**) *VvSUC27*-OE lines Arabidopsis seeds. Magnification of the seeds for each transgenic line (right) highlight changes in seed morphology; c, cotyledon; h, hypocotyl; and r, embryonic root or radicle. Bars = 100 μm. The white arrows indicate the major and minor axes of a successfully segmented seed. (**E**) The seed length, width, and area were compared using scanning electron microscopy images. The seed length and width were measured as major and minor axes (white arrows in **A**), respectively. For seed area, a custom algorithm for ellipse area determined the boundary or contour of the seed (white circle in **A**). For all lines, 20 seeds from each variety were counted and separated (segmented), and the seeds that were touching each other or a piece of debris were omitted from the analysis. Data are expressed as mean ± SD (n = 20). (**F**) Seed germination rate of *VvSUC*-OE lines grown on different sucrose concentrations. Sterilized seeds were placed on Murashige and Skoog (MS) media containing 0%, 3%, 4%, and 6% (*w*/*v*) sucrose. Seed germination was defined and measured as radicle protrusion. Each culture vessel contained 80 seeds. Data are expressed as mean ± SD (n = 3). (**G**) Inhibition rate of seed germination was measured in *VvSUC*-OE seeds and WT grown on MS medium containing 6% (*w*/*v*) sucrose. Different letters indicate significant differences (*P* < 0.05) between each *VvSUC*-OE seeds and WT, as determined by ANOVA followed by Tukey’s test.

To further characterize the functional differences between the three SUTs, homozygous T_3_ *VvSUC*-overexpressing (OE) lines of Arabidopsis were produced and verified by PCR (Figure S2A–C) and RT-qPCR (Figure S2D). The length and width decide the size of the seed, the length reflects the length of the hypocotyl-root axis (major axis), and the width is essentially the sum of the cotyledon and hypocotyl widths (minor axis). Therefore, the size and shape of the exalbuminous seed were determined by the embryo structure and how the embryo folded within [28]. Scanning electron micrographs showed that the embryo folded within *VvSUC11*- and *VvSUC12*-OE seeds (Figure 1B,C) were basically normal compared with that of wild type (WT) seeds (Figure 1A), whereas *VvSUC27*-OE seeds had significantly deeper folded embryo (Figure 1D). Size differences between the embryos of *VvSUC*-OE and WT seeds are compared in Figure 1E. There was significant difference between the length (i.e., the hypocotyl root major axis) of *VvSUC*-OE and WT seeds; *VvSUC*-OE seeds were shorter than WT seeds and *VvSUC11*-OE seeds were the longest among the *VvSUC*-OE seeds analyzed. The length of *VvSUC12*- and *VvSUC27*-OE seeds was similar to each other. Among all the seeds evaluated, in the matter of width, *VvSUC12*-OE seeds were the largest (e.g., cotyledon and hypocotyl widths) and *VvSUC27*-OE seeds were the smallest. The *VvSUC27*-OE seeds were the smallest and WT seeds were the largest. The inhibitory effects of exogenous sucrose on germination in the different *VvSUC*-OE lines indicated that high sucrose concentrations resulted in a lower germination rate of all the seeds, although this inhibition attenuated over time (Figure 1F). The inhibition rate of each *VvSUC*-OE line was compared with that of WT on 6% sucrose-supplemented medium, and the results indicated that *VvSUC11*-OE lines had a similar inhibition rate, while *VvSUC12*-OE and *VvSUC27*-OE lines had weaker and stronger germination inhibition rates than that of WT, respectively (Figure 1G).

### 2.3. VvSUC-OE Lines Have Developmental Phenotypes When Grown on Sucrose

**Figure 2:**
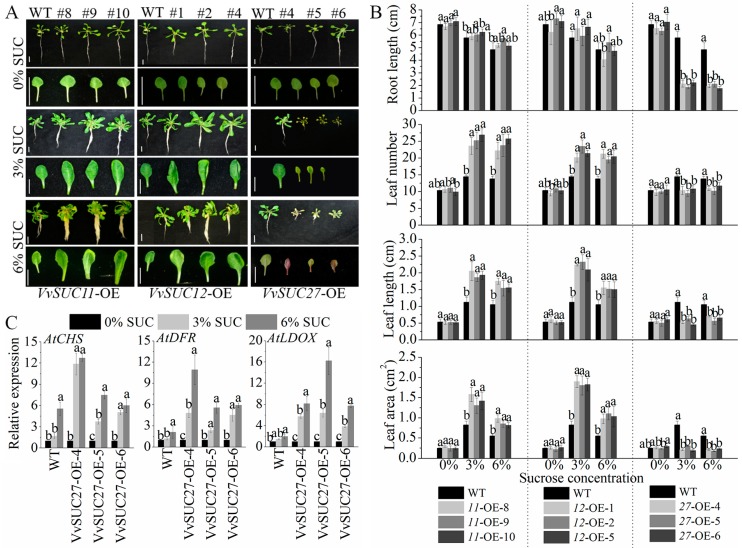
Phenotypes of 4-week-old *VvSUC*-OE Arabidopsis grown in medium supplemented with different sucrose concentrations. (**A**) The phenotype differences of whole seedlings and leaves of *VvSUC11*-, *VvSUC12*-, and *VvSUC27*-OE lines, compared with those of WT. Bars = 1 cm. (**B**) The phenotype differences in the root length, leaf number, leaf length, and leaf area between *VvSUC*-OE lines and WT. Data are expressed as mean ± SD (n = 5). Different letters indicate significant differences (*P* < 0.05) between each *VvSUC*-OE line and WT at one sucrose concentration, as determined by ANOVA followed by Tukey’s test. (**C**) The expression levels of *AtCHS*, *AtDFR,* and *AtLDOX* in *VvSUC*-OE grown on MS media supplemented with 0%, 3%, and 6% sucrose. The cDNA from 4-w-old leaves was isolated and tested for the presence of *Actin*, which served as the internal control for gene expression. Relative gene expression in each *VvSUC*-OE line was calculated by comparing the expression values from seedlings grown on sucrose-supplemented media with those of the control medium, which was set to one. Data are expressed as mean ± SD (n = 3). Different letters indicate significant differences (*P* < 0.05) among different sucrose concentrations, as determined by ANOVA followed by Tukey’s test.

The seeds of the *VvSUC*-OE lines were sterilized and germinated on Murashige and Skoog (MS) medium supplemented with 0%, 3%, and 6% sucrose under in vitro conditions, and then 4-week-old seedlings were harvested to analyze the influence of sucrose on *VvSUC*-OE plant growth (Figure 2). There was no significant difference in phenotypes between the *VvSUC*-OE lines and WT grown on no sucrose-supplemented MS medium (Figure 2A,B). The *VvSUC11*- and *VvSUC12*-OE lines had a stronger developmental phenotype (more number,longer and larger leaves) than that of WT on both sucrose-supplemented MS media (Figure 2A,B). As the sucrose concentration increased to 6%, the roots of *VvSUC11*- and *VvSUC12*-OE lines had a more number of lateral roots, but their root length and leaf number were unchanged; however, the leaf length and area decreased in these lines compared with themselves on medium with 3% sucrose (Figure 2A,B). In *VvSUC27*-OE lines, the *VvSUC27*-OE lines showed the shorter leaves and roots, less number of, and smaller leaves on both sucrose-supplemented MS media; besides, the higher sucrose concentration did not influence root length or leaf number, length, and area; however, the leaf color was purple on 6% sucrose MS medium (Figure 2A,B). It has been reported that sucrose signaling can specifically induce anthocyanin accumulation [29]. Because the purple coloration may indicate the synthesis and accumulation of anthocyanins, we investigated the expression of the main anthocyanin biosynthetic genes [30], *AtCHS* [31], *AtDFR* [32], and *AtLDOX* [33] and found that these genes in the *VvSUC27*-transformants were induced, especially on 6% sucrose-supplemented MS medium (Figure 2C).

### 2.4. Dry Weight, Soluble Sugar Concentrations, and Endogenous Plant Growth Hormone Levels Were Altered in the VvSUC-OE Lines

**Figure 3:**
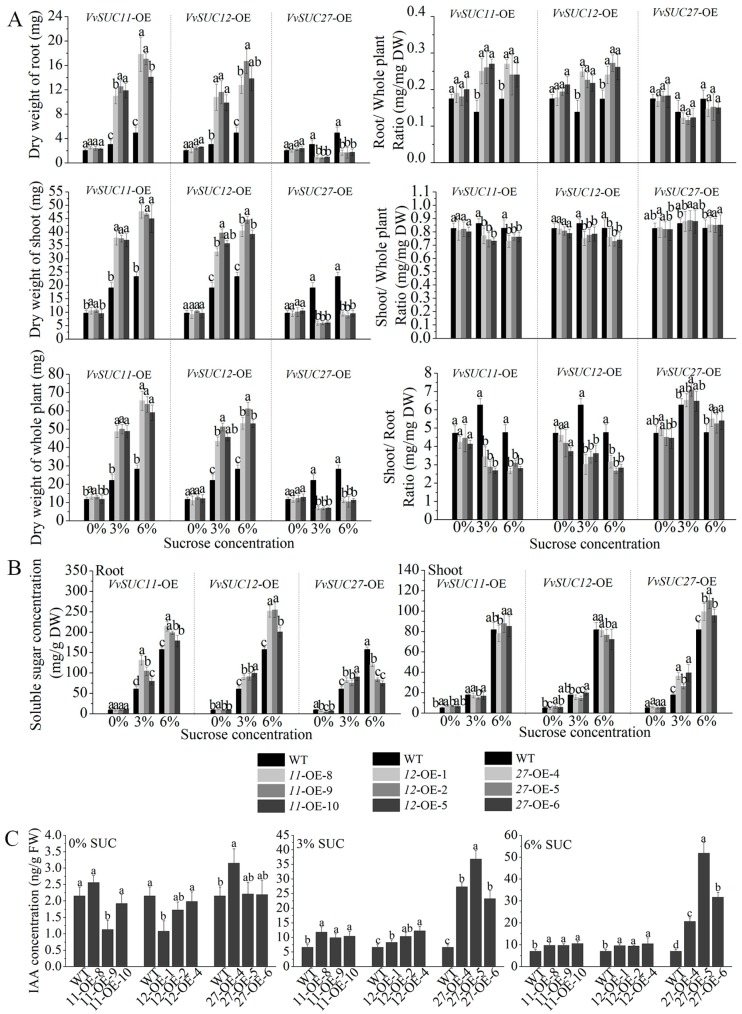
Dry weight, soluble sugar concentration, and endogenous plant auxin levels in 4-week-old *VvSUC*-OE Arabidopsis grown in media supplemented with different sucrose concentrations. (**A**) The dry weight of roots, shoots, and whole plants and the dry weight ratios for root/whole plant, shoot/whole plant, and shoot/root of *VvSUC11*-, *VvSUC12*-, and *VvSUC27*-OE lines, respectively. DW = dry weight. (**B**) The soluble sugar concentrations in the roots and shoots of *VvSUC*-OE lines. (**C**) The indole-3-acetic acid (IAA) concentration in the seedlings of *VvSUC*-OE lines. FW = fresh weight. Data are expressed as mean ± SD (n = 3). Different letters indicate significant differences (*P* < 0.05) between each *VvSUC*-OE line and WT, as determined by ANOVA followed by Tukey’s test.

The *VvSUC*-OE lines had different growth phenotypes after four weeks of cultivation in response to sucrose concentration. The *VvSUC11*- and *VvSUC12*-OE lines developed thicker leaves and roots on sucrose-supplemented media than those of WT plants. Although the *VvSUC27*-OE lines showed inhibited phenotypes, the leaves and roots were thicker than those of WT (data not shown). We measured the dry weight of 4-week-old transformants and found that compared with that of WT, *VvSUC11*- and *VvSUC12*-OE lines had significantly improved growth on sucrose-supplemented media. Additionally, we observed increased root, shoot (leaves and stems), and whole plant weight of the *VvSUC*-OE lines on both 3% and 6% sucrose-supplemented media compared with those of WT (Figure 3A). The thicker leaves and roots of the *VvSUC27*-OE lines did not contribute to increased dry weight; the root, shoot, and whole plant dry weight were significantly less than those of WT. The dry weight ratio was not significantly different between the *VvSUC*-OE lines and WT on media without sucrose. The dry weight of both *VvSUC11*- and *VvSUC12*-OE lines revealed higher root/whole plant ratio, but lower shoot/whole plant ratio, indicating a decreased shoot/root ratio compared with that of WT grown on 3% and 6% sucrose-supplemented media. Interestingly, the dry weight of roots or shoots/whole plant ratios of the *VvSUC27*-OE lines were similar to those of WT; however, the shoot/root ratio was marginally higher in 3% and 6% sucrose-supplemented media, indicating a more severe developmental inhibition of root than shoot growth in the *VvSUC27*-OE lines.

We then verified whether soluble sugar concentrations were affected by *VvSUC*-OE. Compared with that of WT, increased sucrose concentrations could only be observed in the roots of *VvSUC11*- and *VvSUC12*-OE lines grown on 3% and 6% sucrose-supplemented media (Figure 3B). The soluble sugar concentrations also increased in the roots of *VvSUC27*-OE lines grown on 3% sucrose-supplemented medium but decreased in plants grown on 6% sucrose-supplemented medium; however, the soluble sugar concentration in the shoots was higher than that in WT on both 3% and 6% sucrose-supplemented media (Figure 3B).

The endogenous plant growth hormone indole-3-acetic acid (IAA) was also extracted and measured from the seedlings of 4-week-old *VvSUC*-OE lines (Figure 3C). Compared with that of WT, there was no obvious pattern of IAA accumulation in the *VvSUC*-OE lines grown on medium without sucrose; however, the *VvSUC*-OE lines had higher IAA concentration on both 3% and 6% sucrose-supplemented media.

### 2.5. VvSUC-OE Lines Have Developmental Phenotypes When Grown in Soil

**Figure 4:**
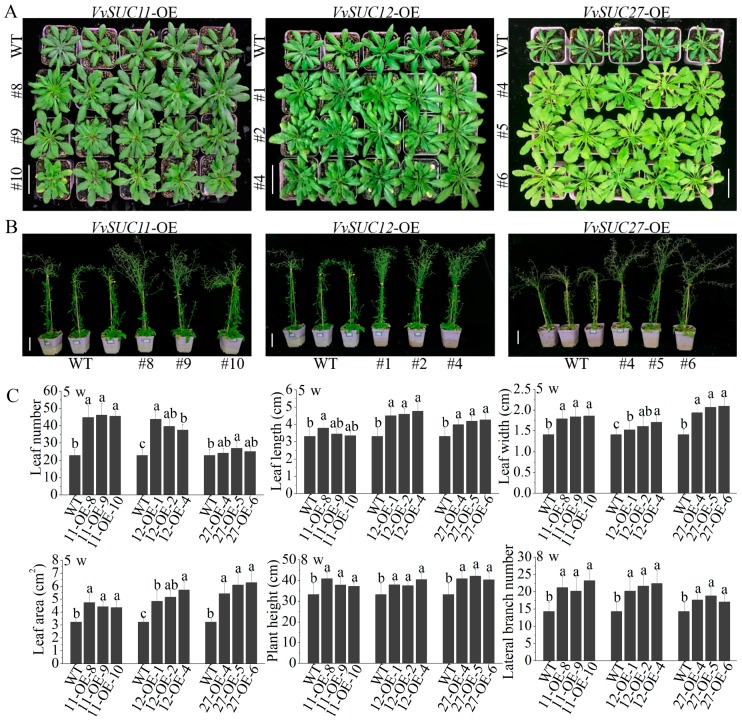
Phenotypes of *VvSUC*-OE Arabidopsis grown in soil. (**A**) The phenotype differences in 5-week-old *VvSUC11*-, *VvSUC12*-, and *VvSUC27*-OE lines, compared with those of WT. Bars = 5 cm. (**B**) The phenotype differences in 8-week-old seedlings of *VvSUC*-OE lines and WT. Bars = 5 cm. (**C**) The phenotypic differences in leaf number, leaf length, leaf width, and leaf area between 5-week-old *VvSUC*-OE lines and WT and the phenotypic differences in plant height and lateral branch number between 8-week-old *VvSUC*-OE lines and WT. Data are expressed as mean ± SD (n = 5). Different letters indicate significant differences (*P* < 0.05) between each *VvSUC*-OE line and WT as determined by ANOVA followed by Tukey’s test.

After characterizing the effects of sucrose on *VvSUC*-OE line growth *in vitro*, the influence of *VvSUC* overexpression in transgenic Arabidopsis was analyzed in plants grown under soil culture conditions (Figure 4). Compared with that of WT, all 5-week-old *VvSUC*-OE lines had enhanced developmental phenotypes, including significantly higher leaf number, length, width, and area, which further confirmed an SUT-dependent effect on plant development (Figure 4A,C). We then used the source leaves to measure sucrose uptake in plants by monitoring the uptake of sucrose-[^14^C]. All the *VvSUC*-OE lines had a higher capacity for sucrose uptake into the source leaves than that of WT (Figure S3). Eight-week-old *VvSUC*-OE lines were noticeably taller and had more numbers of lateral branches than those of WT plants (Figure 4B,C). The yield of the *VvSUC*-OE lines was also analyzed (Table 1), although we did not find a significant improvement in seed number per silique of the *VvSUC*-OE lines; however, significant differences were observed in the silique number between the *VvSUC11*- and *VvSUC12*-OE lines with WT. The *VvSUC11*-, *VvSUC12*-, and *VvSUC27*-OE lines had increased number of siliques than that of WT, with *VvSUC11*- and *VvSUC12*-OE lines presenting the highest number of siliques produced among the transgenic lines. The higher silique number resulted in a higher yield (mg) per plant in the *VvSUC*-OE lines than that of WT Arabidopsis plants.

### 2.6. VvSUC-OE Altered Leaf and Root Structures, Soluble Sugar Concentration, and Endogenous Auxin Levels in Plants Grown in Soil

**Figure 5:**
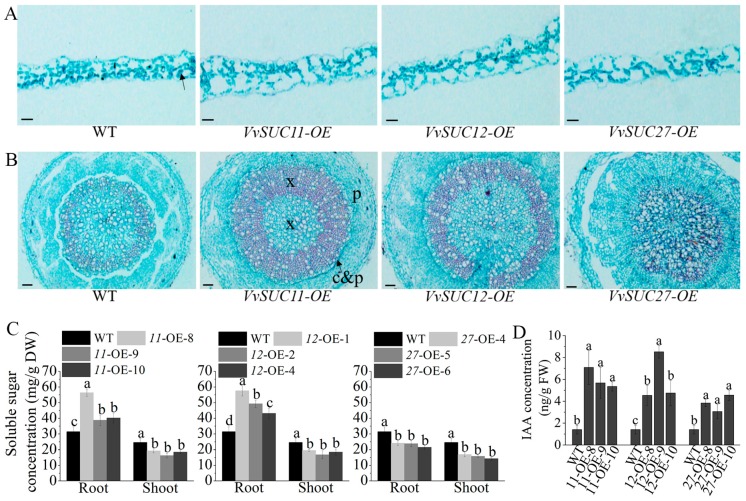
Internal leaf and root morphology, soluble sugar concentration, and endogenous auxin levels in 5-week-old *VvSUC*-OE Arabidopsis grown in soil. (**A**) Cross-cut leaf slices were stained with safranin O/fast green to visualize lignified or keratinized cell walls (red) and cytoplasm or cell walls that contain cellulose (green). Chloroplasts stained dark green and are indicated by black arrowhead. Bars = 100 μm. (**B**) Cross-cut roots slices were stained with safranin O/fast green to visualize lignified or keratinized cell wall (red) and cytoplasm or cell walls that contain cellulose (green). x, xylem; c&p, cambium and phloem; p, periderm. Bars = 200 μm. (**C**) The soluble sugar concentrations in the roots and shoots of *VvSUC*-OE lines. (**D**) The IAA concentration in the seedlings of *VvSUC*-OE lines. Data are expressed as mean ± SD (n = 3). Different letters indicate significant differences (*P* < 0.05) between each *VvSUC*-OE line and WT, as determined by ANOVA followed by Tukey’s test.

The leaves and roots of 5-w-old *VvSUC*-OE lines planted in soil were cross-cut sectioned and analyzed by safranin O/fast green staining. The leaves of *VvSUC*-OE lines were thicker and had fewer chloroplasts than those of WT (Figure 5A). Moreover, the roots were also thicker, especially in the *VvSUC12*- and *VvSUC27*-OE lines (Figure 5B). The overexpression of *SUTs* resulted in enlarged xylem in the *VvSUC11*- and *VvSUC12*-OE lines, whereas the xylem area in the *VvSUC27*-OE lines was reduced compared with that of WT plants (Figure 5B). The soluble sugar concentration was significantly higher in the roots of *VvSUC11*- and *VvSUC12*-OE lines but lower in the *VvSUC27*-OE lines than those of WT (Figure 5C), which was consistent with sugar dose-dependent changes in xylem area. All the *VvSUC*-OE lines had lower soluble sugar concentration in their shoots than those in WT plants (Figure 5C), indicating an increased ability to transport sugar from the source leaves in the three transgenic plants compared with that of untransformed WT. Furthermore, the IAA concentration was also increased in 5-w-old *VvSUC*-OE lines compared with that in WT (Figure 5D).

### 2.7. VvSUC-OE Lines Altered Arabidopsis Drought Resistance When Grown in Soil

**Figure 6:**
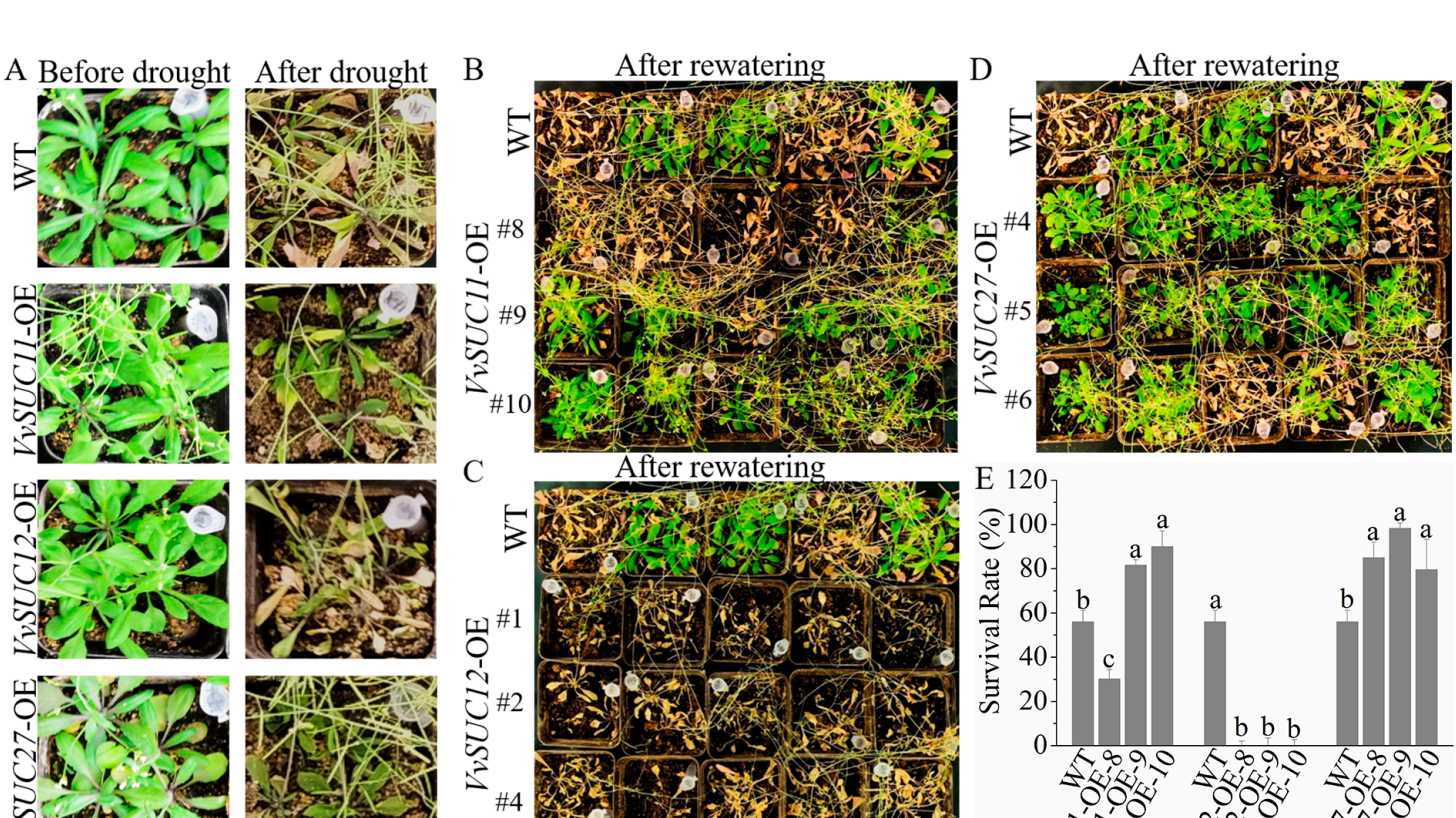
Phenotypic assays of *VvSUC*-OE Arabidopsis under drought stress. (**A**) Phenotypes of *VvSUC*-OE lines before drought and after drought for two weeks. (**B**) to (**D**) Phenotypes of (**B**) *VvSUC11*-, (**C**) *VvSUC12*-, and (**D**) *VvSUC27*-OE lines, which had survived 2 weeks after rewatering. (**E**) Survival rate of *VvSUC*-OE lines after rewatering for two weeks. The survival rates were calculated based upon three biological replicates. Each biological repeat had 20-25 seedlings (four or five seedlings for each pot) for each genotype. Data are expressed as mean ± SD (n = 3). Different letters indicate significant differences (*P* < 0.05) between each *VvSUC*-OE line and WT, as determined by ANOVA followed by Tukey’s test.

To test whether VvSUC regulate drought tolerance, *VvSUC11*-, *VvSUC12*-, and *VvSUC27*-OE lines together with WT were subjected to drought survival assays by withholding water for two weeks and then rewatered for another two weeks as shown in Figure 6. Before withholding water, all the *VvSUC*-OE lines and WT grew normally and healthily (Figure 6A). However, upon encountering drought treatment, most leaves of the plants turned yellow afterwards and were completely dried. After rewatering, the majority of *VvSUC11*-OE lines (excepted for *VvSUC11*-OE-8) (Figure 6B) and *VvSUC27*-OE lines (Figure 6D) rejuvenated, but nearly half of WT and nearly all the *VvSUC12*-OE lines (Figure 6C) did not. So compared with WT, *VvSUC11* and *VvSUC27* could significantly enhanced Arabidopsis drought resistance, whereas *VvSUC12* could obviously decreased Arabidopsis drought resistance (Figure 6E).

## 3. Discussion

### 3.1. VvSUC11 and VvSUC12 Regulate SUT-Dependent Sucrose Transport in Similar Way

SUT family proteins function as key sugar transporters [34]. There are three amino acids sequences encoding the formerly described sucrose transporters named VvSUC11, VvSUC12, and VvSUC27, which have been characterized [23,24,25,26]. In the phylogenetic analysis of VvSUCs, VvSUC11 was characterized as a member of the traditional low-affinity SUT4 subfamily, while VvSUC12 has 66.6% similarity with AtSUC3 and belongs to the SUT2 subfamily (Figure S1). The members of these two subfamilies have been reported to be localized in enucleate SEs of plants [8,17]. The individual roles of VvSUC11 and VvSUC12 were further investigated in transgenic Arabidopsis overexpression lines. The phenotype and germination inhibition rate on high sucrose-supplemented media in *VvSUC11*-OE seeds were similar with those of WT (Figure 1), indicating that overexpression of *VvSUC11* did not influence seed development. However, the germination inhibition rate in *VvSUC12*-OE seeds on high sucrose-supplemented media was lower than that of WT (Figure 1G). Sucrose can induce meristem quiescence as observed in the arrested development of seedlings germinated on high concentrations of sucrose [35]. Hence, VvSUC12 overexpression in Arabidopsis might have inhibited sucrose absorption by the seeds germinated on high sucrose-supplemented media, indicating a regulation role of VvSUC12 on AtSUTs as exogenous sucrose transporters.

Both *VvSUC11*- and *VvSUC12*-OE seedlings produced more and larger leaves as well as rich and thicker roots than those of WT seedlings when grown on sucrose-supplemented media (Figure 2), which resulted in a heavier dry weight of shoots and roots (Figure 3). However, the shoot/root dry weight ratio was lower in both *VvSUC11*- and *VvSUC12*-OE lines than in WT because the root proportion of the ratio increased as sucrose concentration increased. Combined with the significant accumulation of soluble sugar in the roots of both *VvSUC11*- and *VvSUC12*-OE, these results suggest that changes in sucrose transport alter root/shoot development as well as morphology. In higher plant species, the SUT2/SUC3-type transporter is encoded by a single gene, suggesting that SUT2/SUC3-type transporters are essential for plant growth and development. However, AtSUC3 mutants have no recognizable phenotype under standard growth conditions [14], which may act as a putative sucrose sensor in sieve elements. Although VvSUC12 belongs to SUT2/SUC3 subfamilies, we show that the situation is different from AtSUC3 mutants for VvSUC12. Furthermore, AtSUC2 has been reported to be not required for efflux in the transport and release phloem, but its retrieval function likely participates in fine-tuning whole plant carbon partitioning [36]. The changes in the proportion of sugar in the *VvSUC12*-OE shoots and roots were the opposite of this pattern in *AtSUC2* mutants grown in sucrose-supplemented medium [37], which indicated that VvSUC12 might be a similar role in controlling carbon partitioning along the phloem path as AtSUC2. The IAA concentration increased in addition to the stronger phenotype (Figure 3C). These data suggest that VvSUC11 and VvSUC12 are high-affinity SUTs that function to fast load sucrose from the apoplast into the phloem from source when the sucrose concentration is low. This can increase IAA concentrations and encourage root development, and therefore, they can absorb sucrose from the surrounding medium, which can adversely affect photosynthesis, indicated by a decrease in chloroplasts in the leaves.

Both *VvSUC11*- and *VvSUC12*-OE lines grown in soil had similar phenotypes to when they were grown on sterile solid media, including more numbers of, larger, and thicker leaves and a higher IAA concentration than those of WT plants under the same growth conditions (Figure 4 and Figure 5). In addition, the transformants were taller and had more lateral branches, thicker leaves, and more xylem in their roots than those of WT, which resulted in high field (Table 1). These results further verify that VvSUC11 and VvSUC12 could load sucrose produced by photosynthesis in the source leaves. The Arabidopsis *AtSUC2* mutant plants grow poorly, hyperaccumulate carbohydrates in their leaves, and do not produce viable seed [36,38]. As a high-affinity/low-capacity (HALC) system, the SUT1 subfamily is essential for maintaining this sucrose gradient and thereby controlling the rate of phloem sucrose translocation [17]. Although VvSUC11 and VvSUC12 do not belong to the SUT1 subfamily, they are HALC transporters and their high-affinity sucrose transport is especially important for retrieval.

### 3.2. VvSUC27 Shows the Strong Sucrose Transport Capacity Along the Phloem Path

VvSUC27 is similar and belongs to the specific high-affinity SUT1 clade in dicots (Figure S1). In a previous study, we determined that VvSUC27 is a plasma membrane protein [27]. Besides, VvSUC27 was expressed in *Nicotiana tabacum* to produce transformants that were sensitive to sucrose and had rapid organ development, especially in roots, when cultured on sucrose-supplemented medium [27]. In this study, VvSUC27 was overexpressed in transgenic Arabidopsis to compare its function with that of the *VvSUC11*- and *VvSUC12*-OE lines. The exterior of *VvSUC27*-OE seeds was shorter and narrower than WT seeds, which resulted in a smaller seed area and embryo (Figure 1). We also observed the inhibition of germination on high sucrose-supplemented media compared with that of WT (Figure 1), consistent with the findings of a previous report [27]. Our results indicate that VvSUC27 has a strong sucrose transport ability that may hardly be regulated by AtSUTs or NtSUTs expressed in the seeds. The inhibitory effects on early seedling development might be due to osmotic stress, a hexokinase-independent mechanism, or changes in ABA signaling [39]. The phenotypes of *VvSUC27* transgenic Arabidopsis were different from those of *VvSUC27*-expressing *N. tabacum* [27]. In this study, the *VvSUC27*-OE lines had inhibited root and leaf growth as well as fewer and smaller leaves; however, IAA concentration in leaves was higher than those of the WT (Figure 2), which directly resulted in lower dry weight and higher shoot/root ratio (Figure 3A). Moreover, the SUTs have the potential to form heterogenous oligomers with other SUTs in transgenic plants [40], which could also contribute to the variation in growth and development of plant varieties. The leaves of *VvSUC27*-OE lines turned yellow and purple on high sucrose-supplemented media and had fewer chloroplasts in the presence of sucrose, especially in the 6% sucrose-supplemented medium (Figure 2A). Sucrose is an effective inducer of anthocyanins in Arabidopsis [29] and AtSUC1 mutants accumulate less anthocyanins in response to exogenous sucrose [41]. Overexpression of VvSUC27 in Arabidopsis resulted in higher sugar concentration in both the shoots and roots of plants grown on 3% sucrose-supplemented medium, but lower accumulation in the roots on 6% sucrose-supplemented medium (Figure 3B), which might indicate that the roots transferred sucrose to the source organ in the presence of excess sucrose. The *VvSUC27*-OE lines had phenotypes similar to those of the *VvSUC11*- and *VvSUC12*-OE lines when grown in soil (Figure 4 and Figure 5), with the exception of its reduced proportion of xylem area and an increased proportion of phloem area. In comparison to WT, these transgenic lines also had less accumulation of soluble sugar, which is consistent with the low-affinity/high capacity (LAHC) transporter, AtSUC4, which also revealed reduced sucrose concentration in overexpressing lines [42]. In contrast to HALC transporters, LAHC transporters are reported to be important for high rates of regulated phloem loading, and LAHC transport may be important under conditions where the loading rates are increased [17]. VvSUC27 as a LAHC transporter may also be important to phloem loading in vine species.

### 3.3. Potential Role for SUTs in Crop Field and Drought Resistance Improvement

Increased demand for primary foodstuffs is outstripping the increase in crop yield and therefore, plant productivity is an important goal for our society [43]. The significantly more numbers of siliques produced and the higher yield per plant (Table 1) in *VvSUC*-OE lines provides a promising gene target for crop improvement. Increasing photosynthetic efficiency could also improve plant productivity [44]; the C_4_ pathway is more efficient than C_3_ photosynthesis because of the higher CO_2_ concentration around the major CO_2_-fixing enzyme ribulose-1,5-bisphosphate carboxylase/oxygenase (Rubisco) [45]. Moreover, engineering traits of more efficient C_4_ photosynthesis into C_3_ crops could ostensibly improve water and nitrogen use efficiencies and increase plant yield in hot and dry environments [46,47]. SUTs play a key role in the allocation and partitioning of photosynthetically fixed carbon in plants and therefore, if sucrose transport efficacy is promoted, the yield of plants could be further optimized. Moreover, high light could enhance photosynthesis, leaf sugar levels, and phloem loading; however, these factors do not influence *SUT* expression directly [48]. Thus, *SUT* overexpression under high light conditions could be an important component of increased plant yield phenotypes. Here, we grew *SUT*-overexpressing Arabidopsis lines under high light condition to confirm that their productivity was higher than that of WT plants. We observed that the transgenic plants produced more siliques with larger size and that they were taller than WT plants (data not shown). Besides, *VvSUC27*-OE Arabidopsis lines could significantly enhanced Arabidopsis drought resistance (Figure 6). Most *VvSUC11*-OE could significantly improve the drought tolerance, excepted for the *VvSUC11*-OE-8 line (Figure 6). Although the *VvSUC11*-OE lines and *VvSUC12*-OE lines showed the similar phonotypes in sterile and soil culture, they possess the different affinity for sucrose. VvSUC11 has a high affinity for sucrose, while VvSUC12 has a middle affinity for sucrose and its long gene contains 14 exons interrupted by 13 introns; such exon/intron organization is also described for AtSUC3 with no sucrose transport activity [23,24,25]. Furthermore, the subcellular localization of VvSUC11 and VvSUC12 may be different, only VvSUC11 in VvSUC has been reported to own putative dileucine-like vacuolar targeting sequence [49]. In our previous studies, *SUC11* and *SUC27* in resistant grapevine could fast and highly response to osmotic stress, while *SUC12* nearly no response [50]. Besides affinity for sucrose, subcellular localization, and transcriptional regulation, the transport activity of sugar transporter is also affected by posttranslational modification. Drought has been reported to induce a possible posttranslational modification for SUT protein in phosphorylation [51], which might cause the different drought tolerance in *VvSUC11*- and *VvSUC12*-OE lines. This study provides a promising method to develop *SUT*-transgenic crops that have high productivity under high light or water shortage culture conditions, such as those common to Xin Jiang, China; Perth, Australia; and the Sahara Desert, Africa.

## 4. Materials and Methods

### 4.1. Phylogeny and Sequence Similarities Analysis

Phylogenetic analysis of *A. thaliana*, *L. esculentum*, and *V. vinifera* sugar transporter protein sequences were performed using neighbor-joining method with 1000 bootstrap replication by MEGA-X (https://www.megasoftware.net/). VvSUCs sequence similarities analysis were performed by DNAMAN (https://www.lynnon.com/).

### 4.2. Plant Material and Growth Conditions

The leaves of *Vitis vinifera* “Thompson Seedless” variety were collected in Shangzhuang (Beijing, China). The leaves were harvested, immediately frozen in liquid nitrogen, and stored at −80 °C.

*Arabidopsis thaliana* ecotype Columbia (Col-0) was used in this study. *Arabidopsis thaliana* seeds were surface-sterilized with successive washes in 75% ethanol and 10% NaClO, and then rinsed 4–6 times with sterile distilled water. The sterilized seeds were sown and grew on MS media containing 0%, 3%, or 6% (*w*/*v*) sucrose and 0.7% (*w*/*v*) agar in 640 mL sterile culture vessels until phenotype observation on seedlings. For Arabidopsis grown in soil, 1/2 MS medium (the half concentration of full MS medium) cultured 2-week-old seedlings were transferred to pots containing vermiculite, black soil, and nutrient soil mixture (1:1:1). All plants were place under 16-h light/8-h dark photoperiod with 3000 lx illuminance in growth chambers at 22 °C.

### 4.3. Generation of Transgene Constructs and Plant Transformation

Gateway-PCR was used to generate *SUT*-overexpressing constructs. The *VvSUC11*, *VvSUC12*, and *VvSUC27* full-length coding regions were amplified from *V. vinifera* “Thompson Seedless” with primers that added the *att* sequences (attB1 and attB2 underlined inTable S2) to the 5′ ends of the PCR products. The primers attB1-For and attB2-Rev were used to extend the attB-adapter sequences. The attB-PCR products were mixed with the pDONR222 vector, 5×BP-Clonase™ Buffer, and BP-Clonase™ (Invitrogen, Carlsbad, CA, USA), and incubated for 4–6 h at 25 °C before proteinase K was added to cease the BP reaction. The entry clones were combined with the pH7WG2D,1 vector, 5×LR-Clonase™ Buffer, and LR-Clonase^™^ (Invitrogen, Carlsbad, CA, USA). The pH7WG2D,1-*VvSUC11*, pH7WG2D,1-*VvSUC12*, and pH7WG2D,1-*VvSUC27* constructs were then confirmed by sequence for plant transformation and the *SUTs* were expressed under the strong *CaMV 35S* promoter in the pDR196 vector.

For Arabidopsis transformation, the SUT constructs described above were transformed into *Agrobacterium tumefaciens* GV3101, which was then used to transform plants using the floral dip method [52]. The seeds harvested from transformed plants were selected for homozygous T_3_ lines, which were used for further phenotypic analyses.

### 4.4. qRT-PCR

Total RNA from transgenic Arabidopsis tissues were isolated as above. Actin from Arabidopsis was used as the internal control to normalize cDNA samples. qRT-PCR was performed using the UltraSYBR Mixture Kit (CWBIO, Beijing, China) in a Rotor-Gene^®^SYBR^®^Green PCR (QIAGEN, Hilden, Germany) with gene-specific primers (Table S3). The threshold cycle for each qPCR with different concentrations of cDNA was determined and compared against the standards (Actin for Arabidopsis).

### 4.5. Scanning Electron Microscopy

Arabidopsis seeds were attached to conducting double-sided adhesive tape (Plano, Wetzlar, Germany). Scanning electron microscopy aluminum specimen holders were covered with the adhesive tape with seeds attached. The specimens were coated with gold for 60 s and observed under the FEI Quanta 200 scanning electron microscope (FEI QUANTA200, Holland).

### 4.6. Anatomical Section Analysis

5-w-old cross-cut leaves and roots of Arabidopsis grown on soil were selected and used for safranin O/fast green staining, as described [53]. The lignin and fiber tissues were stained red and green, respectively, and observed under the Axio Scope A1 (ZEISS, Germany) microscope.

### 4.7. Dry Weight Determination

Four-week-old Arabidopsis plants were harvested from sterile MS media supplemented with sucrose at different concentrations. The roots and shoots (leaves and stems) of each plant were separated and dried at 80 °C until the samples reached a constant weight.

### 4.8. Soluble Sugar Measurements

Soluble sugars were extracted from samples by grinding 5 g of frozen grape berries or 25 mg of dried leaves of *A. thaliana* in 30 mL or 3 mL of ddH_2_O, respectively, and then distilled three times at 100 °C for 30 min. Then, ddH_2_O was added to the samples until the extract solutions reached a final volume of 100 mL for grape berries and 10 mL for *A. thaliana* leaves. Measurements were performed as previously described with some modifications [54,55]. A 0.5 mL aliquot of extract solution was mixed with 5 mL of 4% anthrone solution (dissolved in 88% concentrated sulfuric acid) and incubated in a 90 °C water bath for 15 min. The optical density of both extracts was measured at 620 nm.

### 4.9. Determination of Endogenous Phytohormone Concentration

Endogenous indole-3-acetic acid (IAA) was extracted from Arabidopsis seedlings grown on sterile solid media and in soil as described previously [27,56] and measured using a high-performance liquid chromatography-mass chromatography system (HPLC-MS) (Varian, PaloAlto, CA, USA) in the Beijing Center for Physical and Chemical Analysis.

### 4.10. Drought Stress Treatment

For drought treatment, sterilized *Arabidopsis thaliana* seeds were placed on Semi-MS media as above for 2 weeks. Then the seedlings were transferred to each pot containing same weighed soil. Then the seedlings were watered once per week. After four weeks in soil, water was withheld for 2 weeks. The survival rates were calculated based upon plants that had survived 2 weeks after rewatering from three biological replicates. Each biological repeat had 20–25 seedlings (4 or 5 seedlings for each pot) for each genotype.

### 4.11. Statistical Analyses

One-way analysis of variance (ANOVA) and Tukey’s test were performed to analyze the significance of data using SPSS16.0 (SPSS Corp., Chicago, IL, USA). We used *P* < 0.05 as an indicator of statistical significance.

## Figures and Tables

**Table 1:** Yield of *VvSUC*-OE *Arabidopsis thaliana* plants.

Line	Seeds Number Per Silique	Silique Number	Yield Per Plant (mg)
*VvSUC11*-OE-8	44.71 ± 0.43 ^d^	402.29 ± 31.76 ^a^	339.97 ± 15.83 ^a,b^
*VvSUC11*-OE-9	44.29 ± 0.21 ^d,e^	350.89 ± 15.49 ^a,b,c^	292.75 ± 28.72 ^a,b,c^
*VvSUC11*-OE-10	46.83 ± 0.13 ^a,b^	383.72 ± 28.84 ^a,b,c^	342.39 ± 20.31 ^a^
*VvSUC12*-OE-1	46.31 ± 0.24 ^a,b,c^	381.39 ± 31.07 ^a,b,c^	330.12 ± 23.50 ^a,b^
*VvSUC12*-OE-2	45.89 ± 0.13 ^c^	392.81 ± 28.53 ^a,b^	341.58 ± 35.02 ^a,b^
*VvSUC12*-OE-4	43.92 ± 0.26 ^e^	414.3 ± 37.51 ^a^	345.47 ± 29.17 ^a^
*VvSUC27*-OE-4	47.02 ± 0.25 ^a^	318.39 ± 27.07 ^b,c,d^	282.12 ± 19.72 ^a,b,c^
*VvSUC27*-OE-5	44.74 ± 0.33 ^d^	322.81 ± 22.53 ^b,c,d^	276.58 ± 26.15 ^a,b,c^
*VvSUC27*-OE-6	46.13 ± 0.24 ^b,c^	308.19 ± 19.51 ^c,d^	270.54 ± 21.43 ^b,c^
WT	46.72 ± 0.24 ^a,b^	268.57 ± 16.79 ^d^	244.84 ± 22.91 ^c^

Notes: Mean values and standard errors were calculated from five biological replicates. Significance was determined via comparison with data from the WT. Significance of each parameter was assessed using Student’s t test (Tukey, *P* < 0.05).

## Supplementary Materials

Supplementary materials can be found at https://www.mdpi.com/1422-0067/21/7/2624/s1.

## Abbreviations

SUT or SUC	sucrose transporter
SE	sieve element
LAHC	low-affinity/high capacity
OE	overexpressing
WT	wild type
MS	Murashige and Skoog
DW	dry weight
FW	fresh weight
x	xylem
c&p	cambium and phloem
p	periderm
IAA	indole-3-acetic acid
qRT-PCR	quantitative real-time polymerase chain reaction
