# Supplementary material for: Expression of Sucrose Transporters from Vitis vinifera Confer High Yield and Enhances Drought Resistance in Arabidopsis
Source: Int J Mol Sci. 2020 Apr 9;21(7):2624. doi: 10.3390/ijms21072624 (PMC7177370; doi:10.3390/ijms21072624)
Supplement: Supplementary file 1 [file ijms-21-02624-s001.zip › Supplementary Figures S1-S3.doc]

**Figure S1.** Phylogenetic analysis of SUT proteins from *Arabidopsis thaliana* (*At*), *Lycopersicon esculentum* (*Le*), and *Vitis vinifera* (*Vv*). A phylogenetic tree was constructed via neighbor-joining method with 1000 bootstrap replication.

**Figure S2.** PCR and qRT-PCR verification of Arabidopsis *VvSUC*-OE transformants.

**Figure S3.** Uptake rate of sucrose-[^14^C] in the leaf discs from *VvSUC*-OE Arabidopsis. Data are expressed as mean ± SD (n = 3). Different letters indicate significant differences (P < 0.05) between each *VvSUC-*OE line and WT, as determined by ANOVA followed by Tukey’s test.
