# Supplementary material for: Expression of Sucrose Transporters from Vitis vinifera Confer High Yield and Enhances Drought Resistance in Arabidopsis
Source: Int J Mol Sci. 2020 Apr 9;21(7):2624. doi: 10.3390/ijms21072624 (PMC7177370; doi:10.3390/ijms21072624)
Supplement: Supplementary file 1 [file ijms-21-02624-s001.zip › Supplementary Tables S1-S3.doc]

**Table S1.** Amino acid sequences pairwise comparisons between VvSUC11, VvSUC12, and VvSUC27 from *V. vinifera* “Thompson Seedless”.

|  | VvSUC11 | VvSUC12 |
| --- | --- | --- |
| VvSUC12 | 46.69% | - |
| VvSUC27 | 51.61% | 40.95% |

| **Table S2.** Primers for PCR used in this study. | |
| --- | --- |
| Gene name | Primer Sequence (5’-3’) |
| attB1,2-*VvSUC11* | For：AAAAAGCAGGCTAGATGGCGGTCCCTGGGG  Rev：AGAAAGCTGGGTCTCATGTGTGGACCCTGGATTT |
| attB1,2-*VvSUC12* | For：AAAAAGCAGGCTAGATGCCGGAGACGATGGACG  Rev：AGAAAGCTGGGTCTCACCTCATTGAGGCAGGGA |
| attB1,2-*VvSUC27* | For：AAAAAGCAGGCTAGATGGAGTTAGCCAAGCCTTCTTC  Rev：AGAAAGCTGGGTCTTAAGACGACGGCTGAGTCCTC |
| attB- adapter | attB1-For: GGGGACAAGTTTGTACAAAAAAGCAGGCT  attB2-Rev: GGGGACCACTTTGTACAAGAAAGCTGGGT |

**Table S3.** Primers for qRT-PCR used in this study.

| Gene name | Primer Sequence (5’-3’) |
| --- | --- |
| *VvSUC11* | For；GTCGCCGTTCTGATAATC  Rev：ATTAGCCACATCCAATAACC |
| *VvSUC12* | For：GCAGGCTGAGGTTGAATTG  Rev：GTGGATGAGGAATGGATGAAG |
| *VvSUC27* | For：CGTTATCTCTGTTCGCTGTTATG  Rev：GCTACCGACACCATCATCTG |
| *AtActin* | For：GAATCGCCGACAGAATGA  Rev：TACTGAGGGAGGCCAAGA |
| *AtCHS* | For：GTGTCCTCGTTGTCTGCTCT  Rev：GTTCCAGTCACTTATCCCCA |
| *AtDFR* | For：GATGAAAATGATTGGAGTGA  Rev：TCCGAGTGATAGGAGAGAGC |
| *AtLDOX* | For：TAACTTTCATTCTACACAAC  Rev：GACTCAACACTCACCATCTC |
